# *N*-Acetylglucosamine Promotes Tomato Plant Growth by Shaping the Community Structure and Metabolism of the Rhizosphere Microbiome

**DOI:** 10.1128/spectrum.00358-22

**Published:** 2022-06-06

**Authors:** Jiuyun Sun, Shuhua Li, Chunyang Fan, Kangjia Cui, Hongxiao Tan, Liping Qiao, Laifeng Lu

**Affiliations:** a State Key Laboratory of Food Nutrition and Safety, College of Food Science and Engineering, Tianjin University of Science and Technology, Tianjin, People’s Republic of China; University of Minnesota

**Keywords:** *N*-acetylglucosamine, chitin, rhizosphere microbiome, *Streptomyces*, acetoin, indole-3-acetic acid, tomato, global regulatory networks, microbial ecology, plant-microbe interactions, rhizosphere-inhabiting microbes

## Abstract

Communication between plants and microorganisms is vital because it influences their growth, development, defense, propagation, and metabolism in achieving maximal fitness. *N*-acetylglucosamine (N-GlcNAc), the building block of bacterial and fungal cell walls, was first reported to promote tomato plant growth via stimulation of microorganisms typically known to dominate the tomato root rhizosphere, such as members of *Proteobacteria* and *Actinobacteria*. Using KEGG pathway analysis of the rhizosphere microbial operational taxonomic units, the streptomycin biosynthesis pathway was enriched in the presence of N-GlcNAc. The biosynthesis of 3-hydroxy-2-butanone (acetoin) and 2,3-butanediol, two foremost types of plant growth promotion-related volatile organic compounds, were activated in both Bacillus subtilis and Streptomyces thermocarboxydus strains when they were cocultured with N-GlcNAc. In addition, the application of N-GlcNAc increased indole-3-acetic acid production in a dose-dependent manner in strains of Bacillus cereus, Proteus mirabilis, Pseudomonas putida, and S. thermocarboxydus that were isolated from an N-GlcNAc-treated tomato rhizosphere. Overall, this study found that N-GlcNAc could function as microbial signaling molecules to shape the community structure and metabolism of the rhizosphere microbiome, thereby regulating plant growth and development and preventing plant disease through complementary plant–microbe interactions.

**IMPORTANCE** While the benefits of using plant growth-promoting rhizobacteria (PGPRs) to enhance crop production have been recognized and studied extensively under laboratory conditions, the success of their application in the field varies immensely. More fundamentally explicit processes of positive, plant–PGPRs interactions are needed. The utilization of organic amendments, such as chitin and its derivatives, is one of the most economical and practical options for improving soil and substrate quality as well as plant growth and resilience. In this study, we observed that the chitin monomer N-GlcNAc, a key microbial signaling molecule produced through interactions between chitin, soil microbes, and the plants, positively shaped the community structure and metabolism of the rhizosphere microbiome of tomatoes. Our findings also provide a new direction for enhancing the benefits and stability of PGPRs in the field.

## INTRODUCTION

The rhizosphere represents a critical hot spot for the biogeochemical transformation that underlies the process of soil formation, carbon cycling, and the ultimate productivity of Earth’s terrestrial ecosystems (1). Plants harbor and shape a fascinating diversity of microorganisms by releasing 5 to 21% of the carbon-rich products of photosynthesis into the soil surrounding their roots, known as the rhizosphere microbiome (2). These rhizosphere microbiomes expand the genomic and metabolic capabilities of their hosts, providing or facilitating a variety of essential life-support functions, including nutrient production, immune modulation capabilities, and biotic stress tolerance capacity (3). Among the total microbial diversity associated with plants, a majority of associations involve positive interactions in which microbes serve as biofertilizers and biocontrol agents and can be utilized to promote sustainable crop production (4). Commercially available strains registered as biofertilizers in the Ministry of Agriculture and Rural Affairs of the People’s Republic of China (http://english.moa.gov.cn/), including Bacillus subtilis, Bacillus amyloliquefaciens, Lactobacillus plantarum, Paenibacillus mucilaginosus, Streptomyces microflavus, and others, were primarily isolated from the plant rhizosphere.

Plant growth-promoting rhizobacteria (PGPRs) are plant-positive rhizosphere microbial interactions that play key roles in plant physiology, including the mitigation of biotic and abiotic stresses in plants via the production of auxin, 1-aminocyclopropane-1-carboxylic acid deaminase, cytokinin, and gibberellin and through nitrogen fixation, phosphorous solubilization, and iron sequestration by bacterial siderophores (5, 6). Meanwhile, rhizosphere microbes are also in constant interaction with plants through the biosynthesis of volatile organic compounds (VOCs) that mediate essential functions in distant metabolic communications, allowing plants and the organisms they interact with to tune their growth, development, defense, propagation, and life cycle to achieve maximal fitness (7). Even though the benefits of PGPRs for crop production have been recognized and studied extensively under laboratory conditions, the success of their application in the field varies with climate, soil type, and other environmental factors (8, 9). The seemingly inconsistent performance of microbial inoculants suggests that more explicit and fundamental processes of plant-plant-microbe interaction remain to be elucidated.

Chitin is the second most abundant polysaccharide in living organisms, following cellulose (10). Chitin and chitosan are biodegradable and nontoxic, drawing increasing attention due to their ability to improve soil and substrate quality, plant growth, and plant resilience and to contribute extensively to the development of enhanced and sustainable crop production (10). The addition of chitin and its derivatives can improve the fresh yield weight of lettuce (11), rocket (Eruca sativa Mill.) (12), chickpea (13), potato (14), tomato (15–17), and many other crops (10). However, knowledge of their specific functions in plant growth promotion, cultivation, and agro-environmental sustainability remains limited, restricting their further contribution to yield increase, the predictable activation of plant defenses, the extension of harvest storage life, and the improvement of slow release of optimized nutrients in fertilizers for agricultural products and their microbiomes.

*N*-Acetyl-d-glucosamine (N-GlcNAc), the most abundant carbon-nitrogen bio compound on Earth, is a derivatized glucose monomer found in polymers of chitin, chitosan, and peptidoglycan, which are major constituents of arthropod exoskeletons, filamentous fungi, and bacterial cell walls (16). N-GlcNAc is absorbed and used by microbes, serving as an important source of nutrient and signaling molecules for both catabolic and anabolic purposes in *Bacillus*, *Streptomyces*, and *Candida* species (16–18). Although there is little information about the production of N-GlcNAc in the plant rhizosphere, the application of chitinolytic Streptomyces griseorubens E44G significantly increased the growth and yield parameters of tomato plants (19). Plants also express a multitude of chitinases that have been implicated not only in plant growth and development but also in plant defense and plant-bacterial symbioses (20). In this study, we hypothesized that N-GlcNAc, enzymatically hydrolyzed from chitin, chitosan, or peptidoglycan via chitinases, functions as a key regulatory molecule in interactions between chitin, agricultural plants, and microbial cell walls, shaping the community structure and biochemistry of the rhizosphere microbiome, enhancing plant growth, and activating plant defense systems during plant–microbe collaborative metabolism.

## RESULTS

### Application of N-GlcNAc improved tomato plant growth.

**FIG 1:**
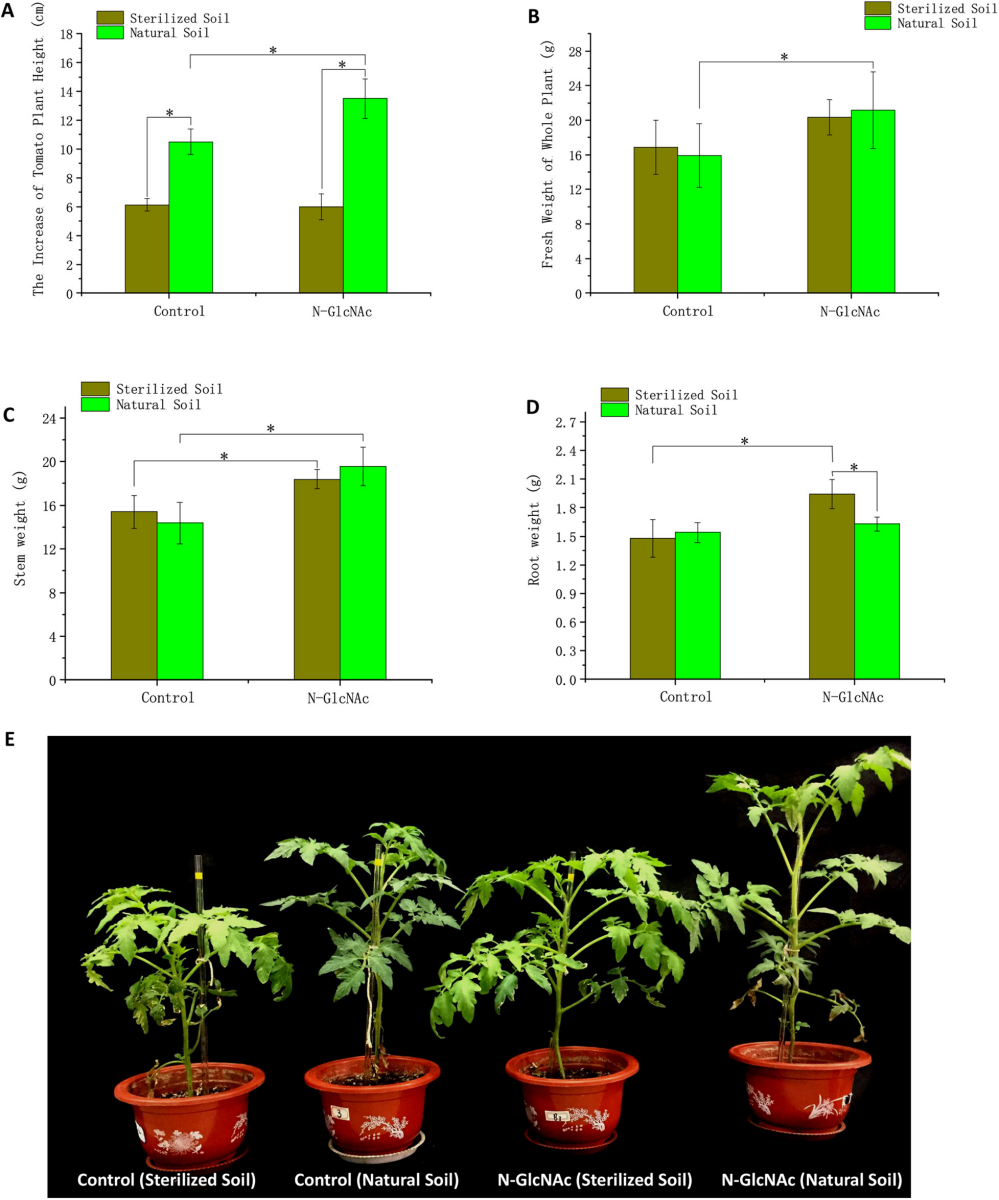
*N*-Acetylglucosamine (N-GlcNAc) soil conditioning increases plant growth. (A) The increase in tomato plant height at 3 weeks with conditioning by N-GlcNAc in natural or sterilized soil. (B) The fresh weight of whole plants. (C) The fresh weight of tomato stems. (D) The dry weight of tomato roots. (E) The growth of plant leaves. Means ± SDs (*n* = 6). One-way ANOVA; *, *P* < 0.05; **, *P* < 0.01.

To verify the hypothesis that N-GlcNAc promotes tomato growth, the growth parameters of Solanum lycopersicum cv. 888F1 plants were obtained for N-GlcNAc-inoculated treatments and controls (Fig. 1). Microorganism-inactivated seeds and sterilized deionized water were used as negative controls. As expected, N-GlcNAc-treated plants produced greater plant height, greater whole fresh weight, and greater stem weight in natural soil (Fig. 1A and C to E). The increase in plant height of N-GlcNAc-exposed plants was 1.29-fold that of plants with water treatments (Fig. 1A). The whole fresh weight of N-GlcNAc-exposed plants was 1.33-fold that of plants with water treatments (Fig. 1B).

However, unlike natural soil, these promotion effects of N-GlcNAc were not observed in the microorganism-inactivation groups. In the microorganism-inactivation groups, seedlings exposed to N-GlcNAc showed a plant height similar to that of the negative-control seedlings (Fig. 1A). The plant height in the microorganism-inactivation group was significantly lower than that in the natural soil group. Enhancement of plant quantity was also not observed in the stems and roots treated with N-GlcNAc solution in natural soil. Interestingly, the N-GlcNAc treatment seemed to show little promoting effect on root weight in the sterilized soil (Fig. 1D).

### Analysis of the effect of N-GlcNAc on the microbial diversity of the tomato rhizosphere microbiome.

Through sequencing the 16S rRNA gene of 18 tomato root samples from the above plant growth promotion tests, we profiled the bacterial community of bulk soil, rhizosphere soil, and the endophytic compartment, which yielded 2,016,900 high-quality, nonchimeric sequences across all samples, with an average of 112,050 sequences per sample. As shown in Table 1, the coverage of all samples was approximately 0.98. The Chao1, Shannon, and Simpson indices showed that the bulk soil and rhizosphere soil microbiomes were richer and phylogenetically more diverse than the endophytic compartment microbiome and that the bulk soil microbial community was less diverse than the rhizosphere. The N-GlcNAc treatment had no significant effect on the alpha diversity of soil microorganisms or the dilution curve. Exogenous application of N-GlcNAc also did not significantly alter the abundance of microbes in the bulk soil, rhizosphere soil, and endophytic compartment.

**TABLE 1:** General information on the sequence and bacterial diversity of tomato plants

Sample ID	No. of OTUs	Chao	Shannon	Simpson	Coverage
Bulk soil					
Control-BS	3,030	3,663.76	8.68869	0.993030	0.992199
GlcNAc-BS	3,122	3,900.73	8.75517	0.992162	0.991738
Rhizosphere soil					
Control-RS	3,454	4,278.92	8.91019	0.992854	0.988873
GlcNAc-RS	3,292	4,101.28	8.47329	0.982556	0.989560
Endophytic compartment					
Control-EC	1,762	2,399.70	5.34212	0.762697	0.984786
GlcNAc-EC	1,540	2,289.01	4.79825	0.691173	0.976369

**FIG 2:**
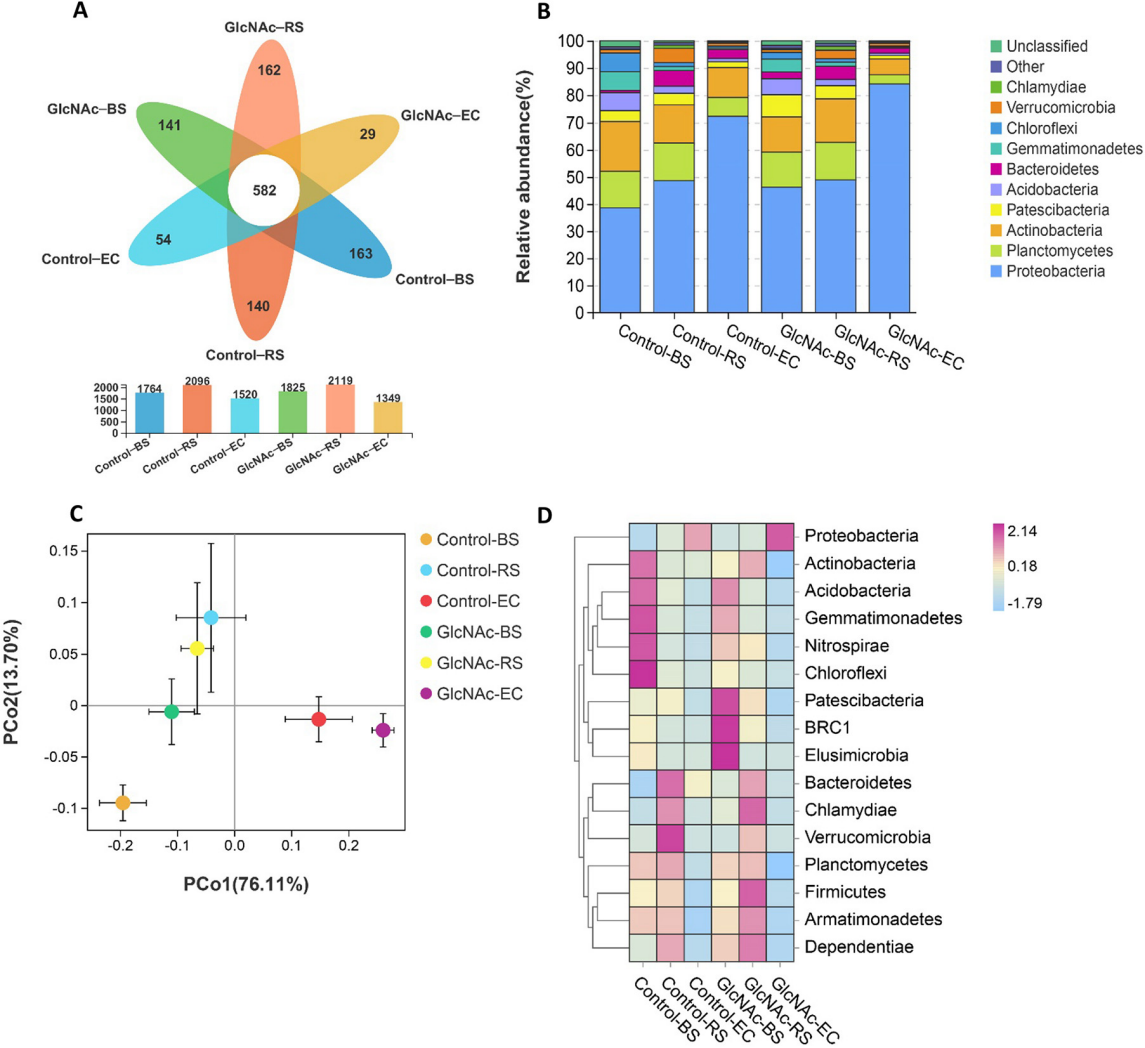
Effects of *N*-acetylglucosamine (GlcNAc) on the soil microbial diversity of rhizosphere soil (RS), bulk soil (BS), and the endophytic compartment (EC) of the tomato root system. (A) Venn diagram showing the number of common and unique species OTUs in samples from different treatments and different soil locations. OTUs with a similarity level of 97% were selected for analysis. (B) Distribution of dominant bacterial groups shown with a stacked bar chart in natural soil and N-GlcNAc-exposed samples at the phylum level. A relative abundance below 2% was classified as “other.” (C) PCoA (principal coordinates analysis) estimates the dissimilarity and similarity of the species diversity of the microbial communities among different treatments. (D) Heatmap presenting the similarities and differences in community composition between natural soil and N-GlcNAc-exposed samples at the phylum level.

Venn analysis (Fig. 2A) showed that all of our tested root samples contained 1,009 common 16S rRNA gene operational taxonomic units (OTUs). N-GlcNAc treatment increased the number of unique 16S rRNA gene OTUs in rhizosphere soil samples while decreasing the number of unique OTUs in bulk soil samples. The rhizosphere soil samples exposed to N-GlcNAc contained 142 unique OTUs that contained relatively abundant *Proteobacteria*, *Actinobacteria*, and *Planctomycetes*. The rhizosphere soil samples contained 95 unique OTUs that were also enriched in *Proteobacteria*, *Actinobacteria*, and *Planctomycetes* (Fig. 2B).

The major components that were driving the differences between samples were quantified using principal coordinate analysis (PCoA) based on Bray–Curtis distances at the OTU level. A clear separation of the microbial community was found and is shown in Fig. 2C. Samples from different compartments and treated with N-GlcNAc were separated, which suggested that the microbial community changed when treated with exogenous N-GlcNAc. The rhizosphere communities in bulk soil and N-GlcNAc-exposed bulk soil also clearly reflected the variance in the microbial community, although the effect of exogenous N-GlcNAc did not reach significance. The bacterial communities in the bulk soil, rhizosphere soil, and endophytic compartment of tomato plant roots exposed to N-GlcNAc are shown in Fig. 2D. The dominant microbial group in the endophytic compartment was *Proteobacteria*, accounting for 84.12%, while the rhizosphere soil sample was dominated by *Proteobacteria*, *Planctomycetes*, *Actinobacteria*, and *Bacteroidetes*, accounting for 48.87%, 15.91%, 13.83%, and 4.86%, and bulk soil samples had more abundant *Acidobacteria*, *Gemmatimonadetes*, and *Chloroflexi* than the rhizosphere soil and endophytic compartments. Moreover, *Proteobacteria* was found enriched in three sample fractions with N-GlcNAc treatment compared to that in untreated samples. In more detail, *Actinobacteria* in untreated rhizosphere soil (10.91%) was less abundant than the sample treated with exogenous N-GlcNAc application (15.91%). In contrast, *Verrucomicrobia* was more abundant in untreated bulk soil (5.19%) but less abundant in N-GlcNAc treatment bulk soil (3.16%). The relative abundance of *Proteobacteria* and *Actinobacteria* in the rhizosphere soil samples of N-GlcNAc-exposed plants was increased by 3.89% and 45.82% that in water treatments, respectively.

### Functional profiles of the rhizosphere microbiome of tomato after N-GlcNAc treatment.

**FIG 3:**
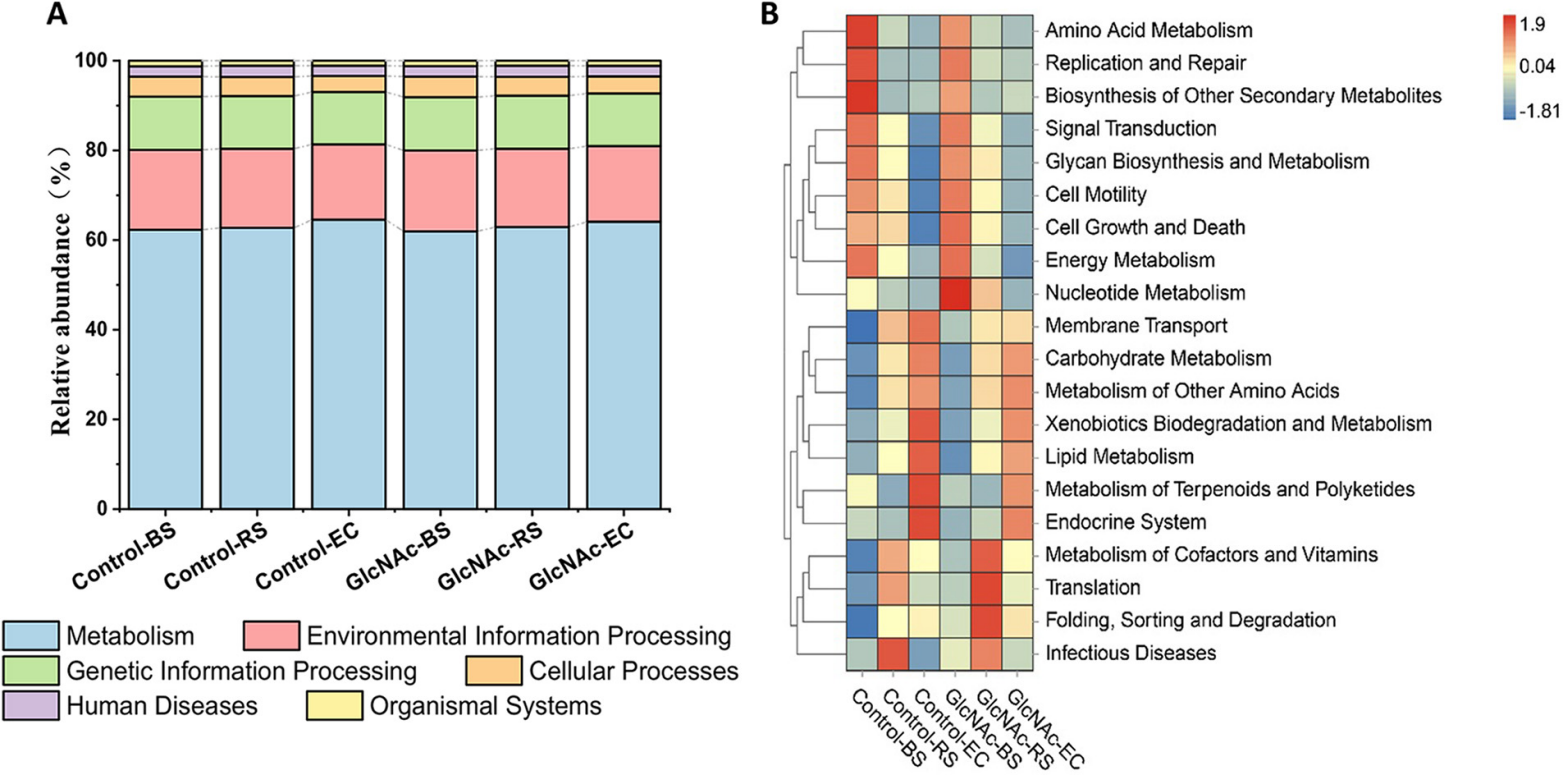
Effects of *N*-acetylglucosamine (N-GlcNAc) on the predicted KEGG pathways of soil microbial diversity of rhizosphere soil (RS), bulk soil (BS), and endophytic compartment (EC) of the tomato root system. (A) Stacked bar chart showing the main predicted pathways at KEGG level 1 by Tax4fun. (B) Tax4fun-derived heatmap and hierarchical clustering comparing the main predicted pathways at KEGG level 2 (relative abundance of >1.0%) of natural soil and N-GlcNAc-exposed samples.

Tax4Fun was used to construct the linear relationship between the SILVA classification and the classification in the KEGG database to predict the function of the microbial community. The abundance of each functional category was calculated according to the OUT abundance. The prediction results (Fig. 3A) showed that at level 1, the predicted pathways for both water-treated and N-GlcNAc-treated rhizosphere soils were basically similar, and the results showed that they were more dominated by metabolism, environmental information processing, and genetic information processing.

To further study the functional information, the metabolic functions of each experimental group were compared at KEGG level 2 (Fig. 3B). The top 5 predicted relative abundances of functions in each sample were amino acid metabolism, carbohydrate metabolism, membrane transport, and signal transduction. Nucleotide metabolism, cofactor and vitamin metabolism, translation, folding, sorting and degradation, glycan biosynthesis and metabolism, and glycan biosynthesis in N-GlcNAc-treated rhizosphere soil were significantly more abundant than those in water-treated rhizosphere soil in control groups, and the abundances of other metabolic functions were also slightly increased.

**FIG 4:**
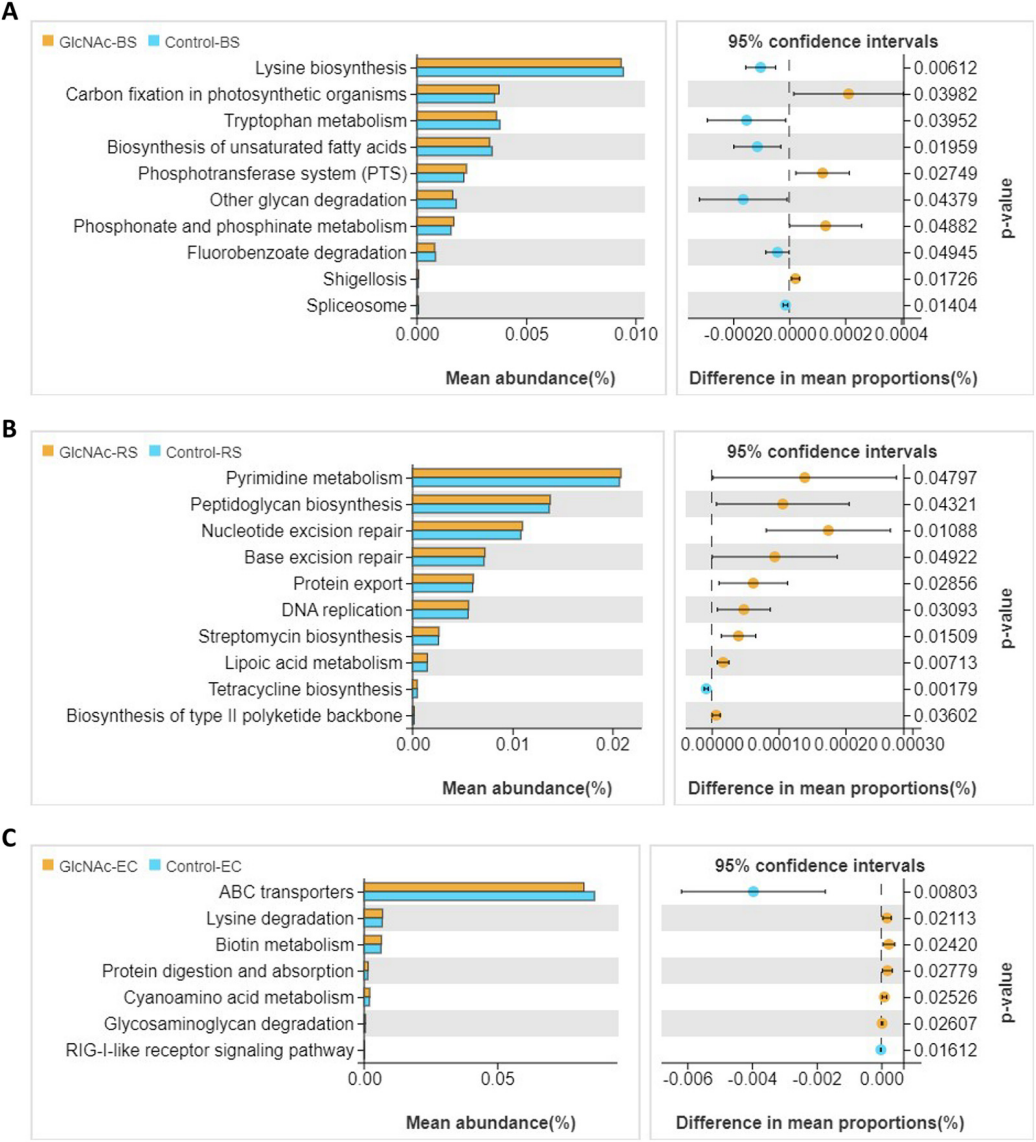
Predicted pathways of soil microbial diversity of rhizosphere soil (RS), bulk soil (BS), and endophytic compartment (EC) of the tomato root system. (A) Statistical differences in predicted functional characteristics between control and *N*-acetylglucosamine (N-GlcNAc)-treated samples in bulk soils (KEGG level 3). (B) Statistical differences in predicted functional characteristics between control and N-GlcNAc-treated rhizosphere soil samples (KEGG level 3). (C) Statistical differences in predicted functional characteristics between control and N-GlcNAc-treated samples in the endophytic compartment (KEGG level 3). Corrected *P* values were calculated using Welch’s test (*P* < 0.05).

Welch’s test of differences between different treatment groups was performed at KEGG level 3. As shown in Fig. 4A to C, the relative abundances of carbon fixation in photosynthetic organisms, the phosphotransferase system, and phosphonate and phosphinate metabolism in N-GlcNAc-treated bulk soil were significantly higher than those in natural bulk soils (*P* < 0.05). In the rhizosphere soil, the addition of N-GlcNAc significantly increased the relative abundances of pyrimidine metabolism, peptidoglycan biosynthesis, nucleotide excision repair, protein repair, and streptomycin biosynthesis. ABC transporters were less abundant in endophytic compartment treated with N-GlcNAc than in control samples, but the relative abundances of lysine degradation, biotin metabolism, and protein digestion and absorption were greater than those in endophytic compartment of water-treated control samples.

### Isolation and functional verification of microbes with growth-promoting ability.

To verify the hypothesis that the improvement of plant growth by N-GlcNAc was based on the recruitment of plant growth-promoting bacteria around the root of tomato, six bacterial strains were isolated from the rhizosphere soil of the tomato plants exposed to N-GlcNAc, named strains Sp-1, Sp-7, Sp-8, Fs-1, Fs-2, and Fs-3. After their pure cultures were obtained, these bacteria were identified through 16S rRNA gene sequencing. The phylogenetic positions of these bacterial isolates were determined by blast searching their 16S rRNA gene sequences against the NCBI database. Phylogenetic analysis showed 96.6% to 99.86% similarity of the 16S rRNA gene sequence of these strains with those of B. cereus, Proteus mirabilis, Pseudomonas putida, S. thermocarboxydus, Streptomyces spinoverrucosus, and Streptomyces mexicanus (see Fig. S1 at https://www.researchgate.net/publication/360756015_Supporting_FigS1_and_Fig_S2_N-Acetylglucosamine_Promotes_Tomato_Plant_Growth_by_Shaping_the_Community_Structure_and_Metabolism_of_the_Rhizosphere_Microbiome_Microbiology_Spectrum).

**FIG 5:**
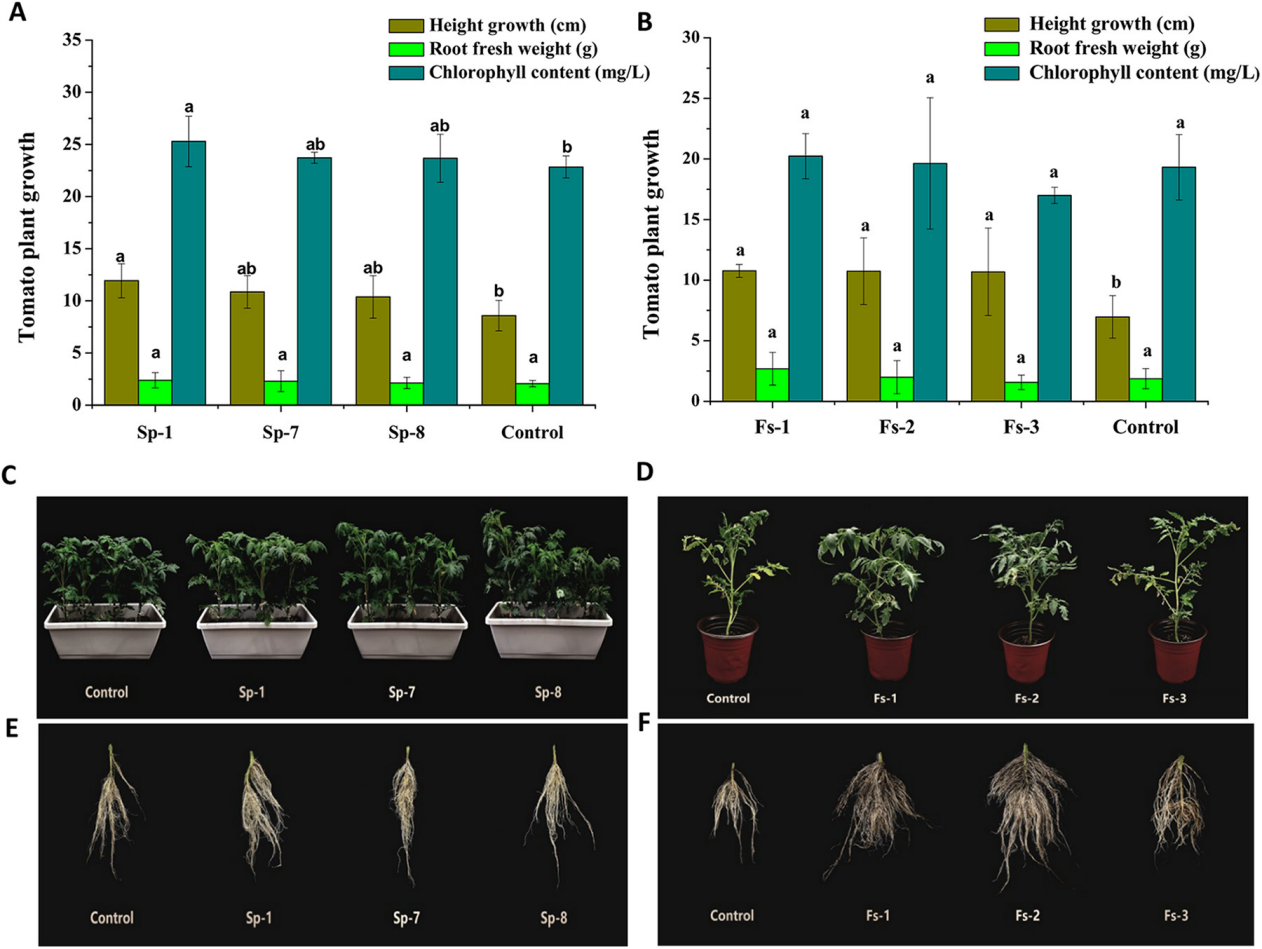
Effects of strains isolated from *N*-acetylglucosamine (N-GlcNAc)-exposed rhizosphere soil on tomato plant growth. (A) Effects of bacterial strains on tomato plant growth height, root fresh weight, and leaf chlorophyll content. (B) Effects of *Actinomycetes* strains on tomato plant growth height, root fresh weight, and leaf chlorophyll content. (C) Effects of bacterial strains on tomato plants. (D) Effects of *Actinomycetes* strains on tomato plants. (E) Effects of bacterial strains on tomato roots. (F) Effects of *Actinomycetes* strains on tomato roots. Means ± SDs (*n* = 6). One-way ANOVA; *, *P* < 0.05; *, *P* < 0.01. Control: natural growth; Sp-1: Bacillus cereus; Sp-7: Proteus mirabilis; Sp-8: Pseudomonas putida; Fs-1: Streptomyces thermocarboxydus; Fs-2: Streptomyces spinoverrucosus; Fs-3: Streptomyces mexicanus.

The overall analyses of plant quantity in the present study showed that the bacterial strains Sp-1 (B. cereus), Fs-1 (*S. thermocarboxydus*), Fs-2 (S. spinoverrucosus), and Fs-3 (*S. mexicanus*) significantly promoted tomato plant height compared with that of the noninoculated control groups (Fig. 5A to D). In B. cereus Sp-1-inoculated plants, a promotion effect on chlorophyll content was also observed compared with that of the control in tomato. All strains showed no promotion effect on fresh weight of tomato roots, while all of them improved root morphology, especially lateral root development (Fig. 5E and F).

### Production of VOCs and indole-3-acetic acid by Bacillus subtilis and *S. thermocarboxydus* Fs-1.

Gas chromatography-mass spectrometry (GC-MS) analysis of VOCs produced by B. subtilis and *S. thermocarboxydus* Fs-1 showed the presence of 32 compounds in LB liquid medium (Table 2). The most abundant compounds among the VOCs produced by B. subtilis included acetone, 2-butanone, and pyrazine, while acetone, 3-buten-1-ol, and 1-butanol were the most abundant compounds in VOCs produced by *S. thermocarboxydus* Fs-1. Analysis of the production of VOCs in B. subtilis and *S. thermocarboxydus* Fs-1 when cocultured with N-GlcNAc revealed an activation in the biosynthesis of plant growth promotion-related substance acetoin and the accumulation of 2,3-butanediol when N-GlcNAc was added to B. subtilis culture medium.

**TABLE 2:** Gas chromatography–mass spectrometry analysis of VOCs produced by Bacillus subtilis and Streptomyces thermocarboxydus Fs-1^*a*^

CAS#	Compound	Retention time (min)	Concn (%)			
			B. subtilis		*S. thermocarboxydus* Fs-1	
			Control	N-GlcNAc	Control	N-GlcNAc
64-17-5	Ethanol	1.565			0.001159	0.003089
67-64-1	Acetone	1.600	0.028984	0.03478	0.008133	0.02093
431-03-8	2,3-Butanedione	2.023		0.012987		0.000607
78-93-3	2-Butanone	2.072	0.001088	0.00021		
565-61-7	2-Pentanone, 3-methyl-	2.129			0.000574	0.001197
141-78-6	Ethyl acetate	2.210	0.000676			
64-19-7	Acetic acid	2.361		0.005851		
78-83-1	1-Propanol, 2-methyl-	2.387			0.000179	0.001926
590-86-3	Butanal, 3-methyl-	2.689				0.000217
96-17-3	Butanal, 2-methyl-	2.812				0.001172
107-87-9	2-Pentanone	3.078	0.001693	0.002126		
547-63-7	Methyl methylpropanoate	3.134			0.000834	0.001777
96-22-0	3-Pentanone	3.333				0.001291
513-86-0	Acetoin	3.511		0.345323	0.000465	0.025696
763-32-6	3-Buten-1-ol, 3-methyl-	3.983			0.002909	0.005157
123-51-3	1-Butanol, 3-methyl-	4.010	0.000258		0.000463	0.001143
137-32-6	1-Butanol, 2-methyl-	4.145			0.002396	0.017882
565-61-7	2-Pentanone, 3-methyl-	4.444	0.000148			0.00036
109-97-7	Pyrrole	4.547	0.0001	0.003768		
71-41-0	1-Pentanol	4.925				0.000852
513-85-9	2,3-Butanediol	5.240		0.000309		
628-32-0	Propane, 1-ethoxy-	5.888				0.000708
921-47-1	2,3,4-Trimethylhexane	6.501		0.002981	0.000831	0.003929
88-09-5	Acetic acid, diethyl-	7.270		0.005677		
2216-34-4	4-Methyloctane	7.994			0.000387	0.001547
110-43-0	2-Heptanone	9.005	0.000363	0.000473		
123-32-0	Pyrazine, 2,5-dimethyl	9.681	0.007156	0.003208		
2371-19-9	2-Heptanone, 3-methyl-	11.096	0.000353		0.000261	
34067-75-9	Cyclopentene, 3-propyl-	11.631		0.001058		
104-76-7	1-Hexanol, 2-ethyl-	14.595	0.001645	0.000851		
821-55-6	2-Nonanone	17.160	9.16E−05	0.000365		
628-99-9	2-Nonanol	17.505	0.000348	0.000383		

a CAS#, Chemical Abstracts Service Registry number.

**FIG 6:**
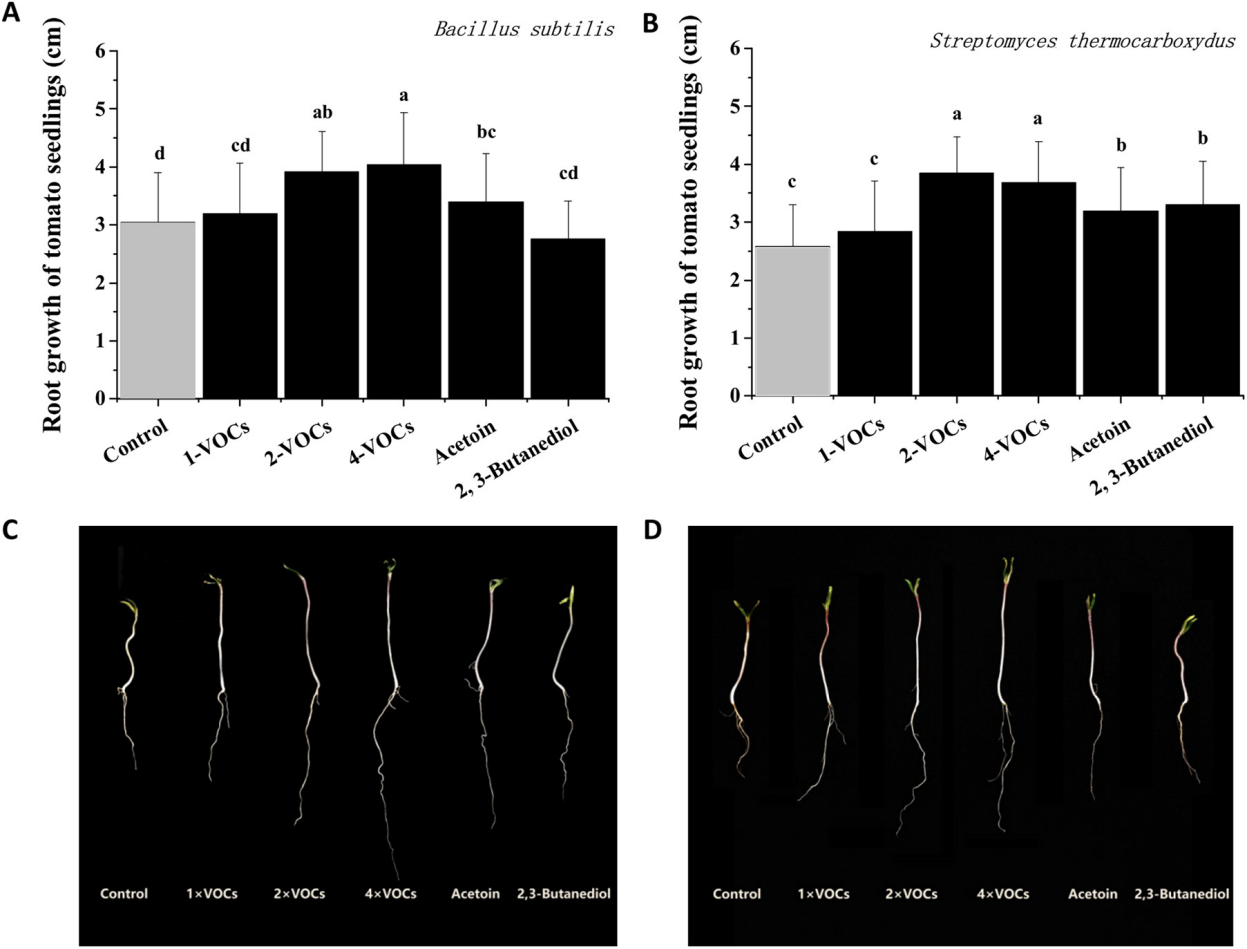
Growth promotion effect of VOCs produced by Bacillus subtilis and Streptomyces thermocarboxydus Fs-1. (A) Effects of the VOCs of B. subtilis on the root growth of tomato seedlings. (B) Effects of the VOCs of *S. thermocarboxydus* Fs-1 on the root growth of tomato seedlings. (C) Tomato root length under the action of VOCs of B. subtilis. (D) Tomato root length under the action of VOCs of *S. thermocarboxydus* Fs-1.

To verify the promotion effect of VOCs of B. subtilis and *S. thermocarboxydus* Fs-1 on the performance of tomato plants, the seedlings were enclosed with the strain and its fermentation broth together during growth for 5 days. The VOCs of both strains at a higher dose, including 2× VOCs and 4× VOCs, significantly increased the growth rate of tomato seedling roots compared with that of the nontreatment water control (Fig. 6A and B). The lengths of the roots exposed to 2× VOCs and 4× VOCs of B. subtilis were 3.91 cm and 4.03 cm, respectively, which were significantly increased by 41.7% and 46.0% compared with those of the water treatments (2.76 cm). Similar results were observed in tomato seeds exposed to *S. thermocarboxydus* Fs-1. The promotion effect of VOCs of *S. thermocarboxydus* Fs-1 on seedling growth also showed a dose-dependent effect. When the seeds were exposed to acetoin, the roots of tomato seedlings were observed to be longer than those of the control and 1× VOCs groups (Fig. 6C and D).

**FIG 7:**
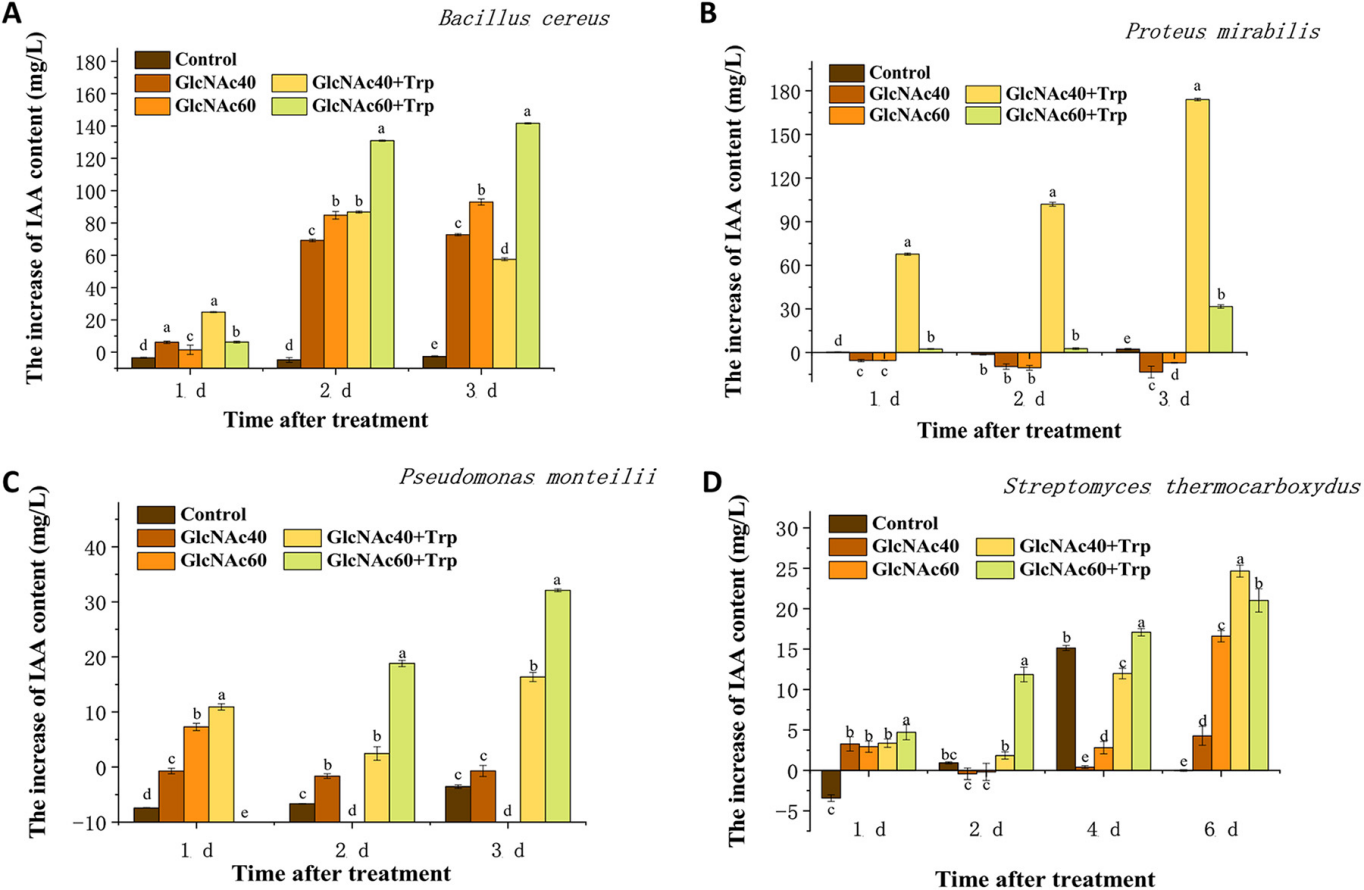
Effect of *N*-acetylglucosamine (N-GlcNAc) on the production of auxin by isolated strains. (A) Changes in auxin content in Bacillus cereus. (B) Changes in auxin content in Proteus mirabilis. (C) Changes in auxin content in Pseudomonas putida. (D) Changes in auxin content in Streptomyces thermocarboxydus. Control: without any addition; GlcNAc40: 40 mmol/L N-GlcNAc; GlcNAc60: 60 mmol/L N-GlcNAc; GlcNAc40+Trp: 40 mmol/L N-GlcNAc and 20 g/L l-tryptophan; GlcNAc60+Trp: 60 mmol/L N-GlcNAc and 20 g/L l-tryptophan.

The auxin IAA produced by four bacterial strains, B. cereus, P. mirabilis, P. putida, and *S. thermocarboxydus*, which were isolated in the present study, was determined at different time intervals postinoculation according to the rate of their growth. IAA, produced by all four strains, significantly accumulated in the presence of N-GlcNAc (Fig. 7). In particular, the increase in IAA content produced by B. cereus reached 92.9 mg/L when cocultured with 60 mmol/L N-GlcNAc in LB medium (Fig. 7A). This ability of N-GlcNAc to activate IAA production was dependent on supplying the exogenous substrate tryptophan to the strains of P. mirabilis and P. putida (Fig. 7B and C). The addition of tryptophan also promoted the accumulation of IAA in strains of B. cereus and *S. thermocarboxydus*. For *S. thermocarboxydus*, the maximum increase in IAA content was 24.7 mg/L, which was recorded when the strain was cocultured with N-GlcNAc at 40 mmol/L and tryptophan at 0.2% (wt/vol) (Fig. 7D).

## DISCUSSION

**FIG 8:**
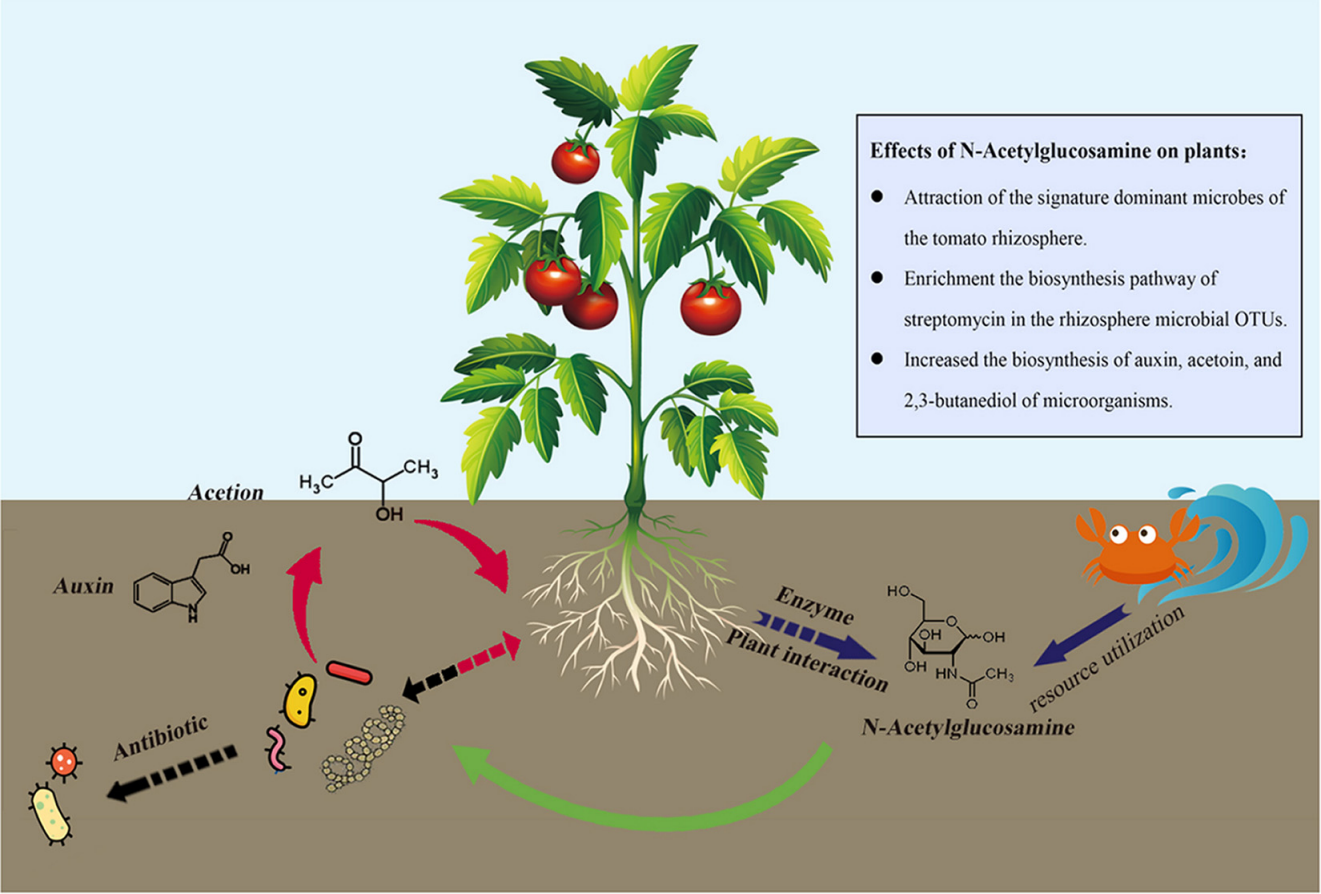
*N*-Acetylglucosamine (N-GlcNAc) may function as a microbial signaling molecule to shape the community structure and metabolism of the rhizosphere microbiome, thereby regulating tomato plant growth and development and plant disease prevention during plant–microbe interactions. N-GlcNAc promoted tomato plant growth via stimulation of signature-dominant microbes of the tomato rhizosphere, such as members of *Proteobacteria* and *Actinobacteria*, and enriched the biosynthesis pathway of streptomycin at the KEGG pathway level. The biosynthesis of 3-hydroxy-2-butanone (acetoin) and 2,3-butanediol, two major types of plant growth promotion-related volatile organic compounds, and indole-3-acetic acid was activated when the five tested microbes were cocultured with N-GlcNAc *in vitro*. Notably, the production of N-GlcNAc through interactions between chitin, microbes, and tomato roots is still a hypothesis based on previous reports and needs further verification.

Here, we first observed that the chitin monomer N-GlcNAc could also improve plant growth by recruiting plant growth-promoting bacteria around the root of tomato plants and activating the production of IAA and growth-promoting VOCs, such as acetoin and butane-2,3-diol, by these bacteria. The biosynthesis of streptomycin was also predicted to be activated in experimental bacterial fermentation broth cultures using LB liquid medium in the presence of N-GlcNAc (Fig. 8). To our knowledge, only one report has focused on the biological function of N-GlcNAc in the plant rhizosphere, and *NOPE1*, a plasma membrane N-GlcNAc transporter, was found to be required for the initiation of arbuscular mycorrhizal symbiosis in both rice and maize (21). The present study found that the monosaccharide molecular N-GlcNAc can serve as a microbial signaling molecule that may be produced by plant chitinase in interacting with the microbe cell wall and chitin, regulating plant–microbe interactions. This process provides an exciting new direction to explore the mechanism by which chitin and its derivatives can function as plant growth-promoting and defense-activating organic amendments in soil.

Plants and their microbiome are reported to function as holobionts (22). Plant roots create specific microbial habitats in the soil and, especially, in their rhizosphere, which comprises a complex assembly of organisms that evolve markedly during plant growth, and rhizosphere microbiomes intimately influence plant health (23). In tomato plants, members of the *Betaproteobacteria* and *Actinobacteria* classes were reported to be highly stimulated and enriched in the rhizosphere and were the dominant, signature microbes of tomato compared with those of cucumber, wheat, and maize (24). In the present study, we observed that the dominant microbes in the rhizosphere zone of tomato were *Proteobacteria*, *Actinobacteria*, and *Planctomycetes*, which was consistent with previous reports for tomato based on 16S rRNA gene clone libraries. The most dominant active bacterial population in the tomato rhizosphere was classified to the orders *Planctomyces*, *Gemmate*, *Streptomyces*, *Devosia*, and *Dokdonella*. Among these orders, *Streptomyces* has attracted the most attention for its antagonistic activities against soilborne fungi and growth promotion ability for its original native plant hosts as demonstrated in previous reports (25). El-Tarabily (26) found that tomato plant growth promotion was most pronounced in the presence of Streptomyces filipinensis no. 15, which was screened from 64 isolates of *Streptomyces* spp. from the tomato rhizosphere. Moreover, six bacterial strains were also successfully obtained in the present study, including B. cereus, P. mirabilis, P. putida, *S. thermocarboxydus*, *S. spinoverrucosus*, and *S. mexicanus*. As expected, these strains showed a plant growth promotion ability, suggesting a general plant-beneficial effect of the members of *the Bacillus*, *Pseudomonas*, and *Streptomyces* orders.

As building blocks of bacterial and fungal cell walls, chitin, chitosan, and N-GlcNAc are often reported to function as signaling molecules, such as pathogen-associated molecular patterns (27, 28). In the present study, the relative abundance of *Proteobacteria* and *Actinobacteria* class members, which were the dominant signature microbes in the tomato rhizosphere, was increased by the application of N-GlcNAc, indicating that N-GlcNAc possessed the ability to enrich the dominant microbes of tomato plants or stimulate plant growth-promoting bacteria around the roots of tomato plants. These findings were consistent with previous reports in which De Tender et al. (29) found that chitin treatment resulted in the increased abundance of plant growth-promoting fungi in the plant root, such as species from the genera *Mortierella* and *Umbelopsis* (30). The relative abundances of the bacterial phyla *Acidobacteria*, *Verrucomicrobia*, *Actinobacteria*, *Bacteroidetes*, and *Proteobacteria* and the fungal phyla *Ascomycota*, *Basidiomycota*, and *Zygomycota* were also significantly changed in chitin-mixed soil of the lettuce rhizosphere (11). Moreover, N-GlcNAc lost its ability to promote tomato plant growth when the rhizosphere and soil microorganisms were inactivated in the present study, proving that the microbiomes were vital in improving tomato plant growth. Considered together, the current results indicate a positive connection between the signaling molecule N-GlcNAc and plant growth-promoting bacteria, providing an explanation for the growth-promoting ability of chitin and its derivatives.

Notably, the streptomycin biosynthesis pathway was also enriched in the N-GlcNAc-treatment groups, indicating that N-GlcNAc may improve the antagonistic activities of tomato rhizosphere microbes against soilborne fungi. This finding was consistent with the previous hypothesis that a high concentration of N-GlcNAc, perhaps mimicking the accumulation of N-GlcNAc after autolytic degradation of the vegetative mycelium, is a major checkpoint for the onset of secondary metabolism (31). These responses are transmitted to antibiotic pathway-specific activators through the pleiotropic transcriptional repressor DasR, a direct repressor for the utilization of N-GlcNAc, which is also required for the proper activation of genes used for chitin degradation (32). In *Streptomyces*, the induction of GlcNAc inactivates DasR and results in the enhancement of antibiotic production (33). Together, these data indicate that the activation of secondary metabolite production of rhizosphere bacteria by N-GlcNAc or chitin and its derivatives may contribute to the increase in disease suppression against fungal soilborne pathogens.

Auxin biosynthesis in bacteria can occur via multiple pathways, including indole-3-acetamide, indole-3-acetonitrile, indole-3-pyruvate, tryptophan side chain oxidase, and tryptamine pathways (34). Auxin IAA plays an important role during interactions between bacteria and their hosts to control plant development in both beneficial and deleterious ways (35). Our results further demonstrated the involvement of IAA in bacteria-tomato plant interactions and establish a positive connection between N-GlcNAc and auxin in all tested strains, including B. cereus, P. mirabilis, P. putida, and *S. thermocarboxydus*. Our observation that the enhancement of tomato plant growth promotion in various orders suggested that the bacteria may share similar signaling pathways to increase their auxin synthesis, and the presence of N-GlcNAc may activate the stress status of bacteria. Broek et al. reported that an increase in auxin levels in host tissue could inhibit plant defenses via stimulation of plant cell growth (36). That is, an increase in IAA content was hypothesized to be crucial for ensuring that PGPRs attained maximum growth promotion during plant–microbe interactions. In plant-associated microorganisms, IAA regulates the expression of genes hypothesized to promote interactions with plants. For example, IAA induces large-scale changes in the transcriptome of Azospirillum brasilense, a PGPR, including upregulation of genes involved in IAA biosynthesis, resulting in a positive feedback loop that reinforces auxin responsiveness (36). However, elevated IAA levels or enhanced auxin signaling could also promote disease development in some plant–pathogen interactions (37). The effect of N-GlcNAc in stimulating plant cell growth during infection by pathogens was unclear, while the application of N-GlcNAc was found to increase IAA content in a dose-dependent manner which inhibited tomato growth when the concentration of N-GlcNAc exceeded 80 mmol/L (unpublished data).

Volatiles mediate the interaction of plants with pollinators, herbivores with their natural enemies, and other plants with microorganisms (7). With increasing knowledge about these interactions, the underlying mechanisms show that rhizobacteria can stimulate root and plant growth through the production of volatiles such as 3-hydroxy-2-butanone (acetoin), 2,3-butanediol, and 2-pentylfuran (38). The current study showed that plant growth promotion-related VOCs were almost undetectable in B. subtilis and *S. thermocarboxydus* Fs-1 fermentation broth cultured in LB liquid medium, while they were enriched in the same fermentation broth in the presence of N-GlcNAc. Surprisingly, the addition of N-GlcNAc activated the biosynthesis of 3-hydroxy-2-butanone and 2,3-butanediol. Glucose metabolism leading to pyruvate allows a diverse group of compounds to be formed, for example, acetate and the growth-promoting VOCs acetoin and butane-2,3-diol (39, 40). Cells avoided the harmful effects of acetate in intracellular carbon flux reduction by overflow of acetoin, the neutral fermentation end product, which played a crucial role in bacterial fitness (22, 41). Hassani et al. (22) found that blocking acetoin overflow pushed more carbon flux from the fructose-6-phosphate to the N-GlcNAc synthesis pathway and greatly improved N-GlcNAc production in B. subtilis. In return, the present study identified a new strategy for stimulating VOCs emission via the utilization of organic amendments, such as N-GlcNAc. Moreover, many bacteria produce acetoin and convert it into pyrazines by condensation reactions, resulting in alkylated and sometimes methoxylated pyrazines, reflecting that the addition of N-GlcNAc might not only interfere with the primary metabolism of the rhizosphere microorganism but also be involved in the alkylation and methoxylation of the metabolites of these cells.

**Conclusion.** In the present study, we demonstrated that N-GlcNAc has a plant growth (plant height)-promoting effect. High concentrations of N-GlcNAc, perhaps mimicking the accumulation of N-GlcNAc after autolytic degradation of the vegetative mycelium, are a major checkpoint for the onset of secondary metabolism and development. The biosynthesis of streptomycin and plant growth promotion-related compounds, such as 3-hydroxy-2-butanone (acetoin), 2,3-butanediol, and the auxin IAA, were activated by N-GlcNAc in the tested bacterial fermentation broth cultured in LB liquid medium, beneficially regulating tomato plant development. Thus, the present study provides a new direction for understanding and utilizing the benefits and stability of PGPRs in the field and reveals a key microbial signaling molecule, N-GlcNAc, used in communications between chitin molecules, agricultural products, microbial cell walls, and plants to shape the microbial community structure and metabolism of the rhizosphere microbiome, thereby simultaneously enhancing plant growth.

## MATERIALS AND METHODS

### Plant materials and growth conditions.

Seedlings of tomato (*Solanum lycopersicum* cv. 888F1) were purchased from Shouhe Seed Industry Co., Ltd., Shandong, China. Tomato seedlings were cultured in soil mixed with steamed and autoclaved perlite at a ratio of 2:1 to improve drainage after transporting to the laboratory at Tianjin University of Science and Technology. Healthy tomato seedlings were selected and cultured in an intelligent artificial climate light incubator (RXZ-600, Ningbo Jiangnan Instrument Factory, China) at 28/23°C (day/night) with a 14-h photoperiod (150 to 200 μmol m^−2^s^−1^ light intensity) and 80% air relative humidity.

For the N-GlcNAc stimulation experiment, the transplanted tomato plants were watered with 50 mL of 40 mmol/L N-GlcNAc (FA72877, Heowns Biochem Technologies. LLC, TianJin, China) solution 3 times per week; sterile distilled water was used as the control, and both these two treatments were defined as natural soil groups. Each treatment was performed twice with 8 tomato plants per treatment. The growth status index of the tomato plants was measured at the end of the experimental procedure. To verify the roles of microorganisms in N-GlcNAc stimulation experiment, a microbially inactivated rhizosphere environment was created via sterilizing the seedlings with 75% ethanol for 3 times at 1-min time intervals and rinsed with sterile distilled water each time during the seedling transfer process. The soil was sterilized at 121°C for 20 min to reduce the activity of the tomato rhizosphere microorganisms, defined as sterilized soil groups.

### Soil sampling and rhizospheric microorganism collection.

After 21 days of growth, the tomato plants were harvested to sample their rhizosphere and endophytic microorganisms as described below in natural soil groups (42). The roots were shaken to remove loose soil until only soil within 1 mm of the root surface remained. The roots were placed in a clean and sterile 250-mL conical tube containing 50 mL of distilled water. Rhizospheres were separated from the roots by shaking the root system 3 times in distilled water at 180 rpm for 20 min. The resulting turbid solution was collected and centrifuged in 2 steps to form tight pellets (averaging 1 g), defined as rhizosphere soil samples (GlcNAc-RS or control-RS). Bulk soil samples (GlcNAc-BS or control-BS) were taken by discarding the top 1 cm of soil from pots and scooping 1 g of mixed soil into a 2-mL tube. Roots were rinsed in 75% ethanol for 1 min and then treated 3 times with sterile distilled water for 3 min to isolate the endophytic compartment microbial community (GlcNAc-EC or control-EC). All bulk soil, rhizosphere soil, and endophytic compartment samples were directly frozen and stored at –80°C until DNA was extracted.

Rhizosphere bacterial strains were isolated from the healthy tomato roots of the GlcNAc-RS groups in natural soil. Soil suspensions (200 μL) of GlcNAc-RS groups diluted to 10^−4^ to 10^−7^ times with sterile water were spread on lysogeny broth (LB) or Gause’s synthetic agar medium (soluble starch, 20.0 g; KNO_3_, 1.0 g; K_2_HPO_4_, 0.5 g; MgSO_4_·7H_2_O, 0.5 g; NaCl, 0.5 g; FeSO_4_, 0.01 g; agar, 10.0 g; distilled water, 1,000 mL) at 28°C for 3 or 15 days, respectively. The initial characterization of bacterial and actinomycete strains was performed according to their colony morphology. For identification of the bacterial strains, a bacterial genomic DNA extraction kit (Axygen Biosciences, Union City, CA, USA) was used to extract genomic DNA. The universal primers 27F: 5′-AGAGTTTGATCMTGGCTCAG-3′ and 1492R: 5′-TACCGGYTACCTTGTTACGACTT-3′ were used to amplify the nearly full-length fragment of 16S rRNA gene, and PCR was performed on the 16S rRNA gene fragment (94°C for 3 min; 24 cycles of 94°C for 30 s, 54°C for 30 s, and 72°C for 1 min; and a final extension at 72°C for 10 min). The PCR products were detected by 1.0% agarose gel electrophoresis and then sequenced in both directions by a DNA analyzer (Applied Biosystems, Foster City, CA, USA). The 16S rRNA gene sequence obtained by sequencing was compared with the bacterial strains reported in GenBank (http://www.ncbi.nlm.nih.gov) for homology, and the sequence with the highest homology was found for similarity analysis using MEGA 7.0. A phylogenetic tree was constructed to determine the taxonomic status of strains (43, 44).

To verify the promotion effect of the rhizosphere bacteria strains in tomato plant growth, all isolated strains were cultured in LB liquid medium and obtained by centrifugation. The strains were resuspended with 100 mL of sterile distilled water to water the tomato seedlings roots twice during the day 21 test, and the height and condition of plant growth were recorded at that time.

### Genomic DNA extraction and Illumina HiSeq 2500 sequencing.

Microbial genomic DNA in different soil groups was extracted using HiPure soil DNA kits (Magen, Guangzhou, China) according to the manufacturer’s protocols. The V3-V4 region of 16S rRNA gene was amplified by PCR (95°C for 2 min, followed by 27 cycles at 98°C for 10 s, 62°C for 30 s, and 68°C for 30 s, and a final extension at 68°C for 10 min) using the primers 341F, CCTACGGGNGGCWGCAG and 806R, GGACTACHVGGGTATCTAAT, where the barcode was an eight-base sequence unique to each sample (45). PCR was performed in triplicate in a 50-μL mixture containing 5 μL of 10× KOD buffer, 5 μL of 2.5 mM dNTPs, 1.5 μL of each primer (5 μM), 1 μL of KOD polymerase, and 100 ng of template DNA. Then, 2% agarose gel electrophoresis and an AxyPrep DNA gel extraction kit (Axygen Biosciences, Union City, CA, USA) were used to detect and recover PCR products, and an ABI StepOnePlus real-time PCR system (Life Technologies, Foster City, CA, USA) was used to perform quantitative testing. A TruSeq DNA PCR-free sample preparation kit (Illumina, USA) was used for library construction, and an Illumina HiSeq 2500 PE250 was used for sequencing.

### Data collection and bioinformatics analysis.

FLASH software (46) was used to remove adapter sequences and spliced to sequences from raw sequencing data. Sequences of raw tags were filtered by the QIIME (version 1.9.1) pipeline under specific filtering conditions to obtain high-quality clean tags (47, 48). Clean tags were searched against the reference database to perform reference-based chimera checking using the UCHIME algorithm (49). The effective tags were clustered into operational taxonomic units (OTUs) of ≥97% similarity using Usearch (version 7.0) (50). The tag sequence with the highest abundance was selected as the representative sequence within each cluster. Venn analysis was performed with VennDiagram (version 1.6.20) to identify unique and common OTUs among groups. The abundance statistics of each taxon were visualized using Krona (version 2.6). Alpha index comparisons between groups were calculated by Welch’s *t* test. Sequence alignment was performed using Muscle (version 3.8.31), and a Bray–Curtis distance matrix was generated using the GUniFrac package (version 1.0) (51). Principal coordinates analysis (PCoA) of Bray–Curtis distances was performed and plotted with ropls (version1.6.2). KEGG pathway analysis of the OTUs was inferred using Tax4Fun (version 1.0) (52).

### Production of plant growth-promoting VOCs and auxin by bacterial strains.

The bacterial strains *S. thermocarboxydus* Fs-1 (CGMCC no. 21628) and B. subtilis were cultured in LB liquid medium with N-GlcNAc with 40 mmol/L N-GlcNAc for 5 days and 24 h, respectively. The VOCs from these strains were analyzed by a coupled headspace solid-phase microextraction (HS-SPME, 75 μCAR/PDMS, Supelco, USA) and gas chromatography–mass spectrometry (GC–MS, QP 2010 Ultra, Shimadzu, Japan) method during liquid fermentation. GC–MS with an instrument equipped with a capillary column (VF-5 MS, 30 m by 0.25 mm by 0.25 μm, 0.25 μm-thick film, Varian Co.) was used for analysis of the gas composition, and helium with 99.999% purity was used as the carrier gas. The mass spectrometer was operated in electron ionization mode at 70 eV, the temperatures of the ion trap mass separator and the transmission line were kept at 220°C and 280°C, respectively, and mass spectra were obtained with a scan range of 43 to 500 *m/z*. National Institute of Standards and Technology 11 was used as the spectral library, and 2-methyl-3-heptanone (M158862, Aladdin Biochemical Technology Co., Ltd., Shanghai, China) was used as the internal standard.

The growth promotion effect of the VOCs from the bacterial strains *S. thermocarboxydus* Fs-1 and B. subtilis on tomato plants was determined by plant root growth experiments (53). Tomato seeds (MicroTom, PanAmerican Seed, USA) were surface disinfected with a 70% ethanol and 2% (vol/vol) sodium hypochlorite solution. Five treatments were tested in each selected strain in the present study: 1 group of bacterial strains in liquid fermentation broth (1× VOCs, 2× VOCs, and 4× VOCs) that were cocultured with 40 mmol/L N-GlcNAc, acetoin (Aladdin, Shanghai, China), and 2,3-butanediol (Macklin, Shanghai, China). Each treatment was performed with three replications with 2 sterile petri dishes (diameter: 9 cm) containing 15 seeds per replicate in a sterile airtight glass container (40 cm by 20 cm by 15 cm). Acetoin and 2,3-butanediol were used as the positive controls, and they were diluted according to the endogenous content of the microbes in VOCs. The remaining glass tank containing six dishes of tomato seedlings was treated as a control. All containers were placed in an intelligent artificial climate light incubator at 20°C (day/night) and 50% to 60% air relative humidity for 3 to 5 days, and the root length of each tomato seedling was measured at the end of the experiment. The auxin IAA content was monitored by using Salkowshi reagent (54). The supernatant was mixed with Salkowshi reagent at a volume ratio of 1:4 in the dark for 30 min at room temperature. The concentration of IAA in the samples was measured spectrophotometrically at 530 nm using IAA as a standard.

### Statistical analysis.

All statistical analyses were performed with SPSS Statistics 20.0 (SPSS Inc., Chicago, IL, USA). The data are presented as the mean ± standard deviation (SD). The effect of N-GlcNAc on plant growth-promoting characteristics was evaluated using Student’s *t* test. One-way analysis of variance (ANOVA) was performed on functional analysis of microbes with growth-promoting ability and on the production of VOCs and IAA by the microbes, according to Duncan’s multiple range test. Differences were considered significant at a *P* value of <0.05. High-throughput sequencing analysis was performed using OmicShare tools (https://www.omicshare.com/tools), a free online platform for data analysis.

### Data availability.

Raw sequencing files have been deposited in the SRA database (BioProject accession number PRJNA839939).
